# Effect of high NEFA concentration on lipid metabolism disorders in hepatocytes based on lipidomics

**DOI:** 10.3389/fphar.2024.1372296

**Published:** 2024-02-28

**Authors:** Xinyi Fan, Jie Xu, Yuan Hu, Kui Wang, Yiyi Zhao, Jinyin Cai, Xinyuan Zhang, Binghai Pan, Anqi Xu, Yajing Chen, Songhao Liu, Kangfeng Jiang, Xiaobing Li

**Affiliations:** College of Veterinary Medicine, Yunnan Agricultural University, Kunming, Yunnan, China

**Keywords:** fatty liver, nonesterified fatty acids, lipid metabolism disorders, lipidomics, diary cow

## Abstract

**Introduction:** High concentrations of nonesterified fatty acids (NEFA) is the key of characteristic of fatty liver in dairy cows. Therefore, the aim of this study was to investigate the effect of high concentration of NEFA on lipid metabolism in hepatocytes through the lipidomic approach and molecular biology techniques.

**Methods:** Stimulate AML-12 cells with different concentrations of NEFA, observe the cellular lipid accumulation, and select 0.6 mM NEFA stimulation concentration for subsequent experiments. Collect cells for lipidomics analysis.

**Results:** High concentration of NEFA (0.6–2.4 mM) significantly reduced the cell viability in a concentration-dependent manner, indicating that high concentrations of NEFA have lipotoxicity on hepatocytes. In addition, NEFA promoted triglycerides (TAG) accumulation, increased the mRNA expression of the lipogenic molecules SREBP1c and FASN, and decreased the mRNA expression of lipolytic molecules CPT1A and HSL in hepatocytes. Mechanistically, high concentration of NEFA induced lipid metabolism disorders in hepatocytes by regulating metabolic pathways such as glycerol phospholipid metabolism, glycosyl phosphatidylinositol anchored biosynthesis, triglyceride metabolism, sphingolipid metabolism, and inositol phosphate metabolism.

**Discussion:** High concentration of NEFA is lipotoxic to cells, promoting lipid accumulation. LPE (18:2), LPE (18:3), LPE (18:1) via glycerophospholipid metabolism, glycosylphosphatidylinositol (GPI)-anchor biosynthesis, glycerolipid metabolism, sphingolipid metabolism, and inositol phosphate metabolism, indicating their potential regulation role in the pathogenesis of fatty liver.

## 1 Introduction

Fatty liver is a metabolic disorder that affects dairy cows during the periparturient period, characterized by the accumulation of excess lipids in the liver due to an imbalance between lipid intake and oxidation (Bobe et al., 2004). In the perinatal period, the dietary intake of dairy cows is inadequate to meet the energy demands necessary for lactation, leading to a negative energy balance (NEB) (Herdt, 2000). During NEB, TAG are mobilized from adipose stores (Dole, 1956; Drackley et al., 1991) and then hydrolyzed into NEFA and glycerol (Locher et al., 2011). The released NEFA can be utilized for energy production and fat synthesis (Adewuyi et al., 2005). However, the hepatic uptake of NEFA during NEB exceeds its oxidative capacity, resulting in hepatic lipid accumulation and the development of fatty liver (Roberts et al., 1981; Reynolds et al., 2003; Martens, 2023).

In recent years, multiple studies have demonstrated the involvement of the sterol regulatory element-binding transcription factor (SREBF-1c) in modulating triglyceride and cholesterol levels (Guo et al., 2014; Vargas-Alarcon et al., 2019). SREBF-1c regulates the expression of genes involved in the biosynthesis of fatty acids, phospholipids, and triglycerides (Shimano et al., 1997). SREBP-1c is a transcription factor important in the regulation of lipogenic genes (Yang et al., 2001), and its downstream target FASN is a key enzyme in *de novo* fatty acid synthesis (Horton et al., 2002; Rodríguez-Cruz et al., 2012; Chen et al., 2018). Hormone-sensitive lipase (HSL) is the major rate-determining enzyme in fat cell lipolysis (Fredrikson et al., 1981). CPT1A plays an important role in using fatty acids as an energy source (Patsoukis et al., 2015). CAPT1A and HSL are commonly utilized as representative genes for assessing lipid levels through lipolysis (Hu et al., 2020; Tardelli et al., 2020; Oh et al., 2021). The study chose to investigate the expression of the well-known genes SREBP-1 and FASN involved in lipid synthesis, as well as the genes CAPT1A and HSL related to lipid decomposition, to evaluate lipid accumulation.

Lipids are a group of molecules with various important cellular functions, including energy storage, signaling, and serving as essential components of cellular membranes (Orešič et al., 2008). The alteration of lipid metabolism represents a crucial step in the development and progression of fatty liver disease (Onorato et al., 2021). Among other techniques, recent advancements in mass spectrometry have introduced lipidomics into translational medicine and research (Ma and Fernández, 2022; Salvador et al., 2022). Lipidomics is an emerging and effective method for studying intact lipids in biological systems, which contributes to comprehensively understand the biochemical mechanism of lipid metabolism (Long et al., 2020). Lipidomics relies heavily on analytical chemistry tools, techniques, and principles to analyze lipid structure, abundance of individual molecular species, as well as their cellular functions and interactions. This comprehensive approach enables the identification of dynamic changes in lipids during cellular perturbations. Therefore, lipidomics plays a crucial role in elucidating the pathogenesis of lipid-related diseases such as fatty liver, by detecting and quantifying alterations in cellular lipid signaling, metabolism, transport, and homeostasis (Han and Gross, 2021). Consequently, studying lipid homeostasis is essential for a comprehensive understanding of the pathogenesis of fatty liver.

An increasing number of studies indicate a close relationship between NEFA and lipid metabolism disorders in hepatocytes (Rukkwamsuk et al., 1999; Li et al., 2012; White, 2015). However, the specific mechanism by which NEFA affects lipid metabolism disorders in hepatocytes remains unclear.

## 2 Materials and methods

### 2.1 Cell culture

The AML-12 cell line was acquired from the Cell Bank of the Chinese Academy of Sciences. The cells were cultured in F12 medium (Gibco) supplemented with 10% fetal bovine serum, 1% ITS (Gibco), and 1% dexamethasone. Culturing of cells was routinely performed in a humidified atmosphere at 37°C with 5% CO_2_.

### 2.2 Cell viability assay

AML-12 cells (10^4^ cells/well) were seeded into 96-well plates and then exposed to varying concentrations of NEFA (0, 0.3, 0.6, 1.2, 2.4 mM) for 12 h. Cell viability was assessed using the CCK8 analysis kit (Bioss). Subsequently, 10 μL of CCK-8 solution was added to each well, followed by incubation for 4 h, and the optical density (OD) was measured at 450 nm using a microplate reader.

### 2.3 TAG content assay

AML-12 cells were stimulated with NEFA for 12 h. The stimulated cells were collected and rinsed three times in PBS. The cell lysate was incubated on a shaker at room temperature for 20 min, followed by collection of the cells using a scraper and transfer to a centrifuge for ultrasonic pulverization. Subsequently, reagents were added according to the manufacturer’s instructions (NJJCBIO), and measured 450 nm OD of each well using microplate reader. The protein concentration of each sample was determined using BCA, and the total amount of triglycerides was calculated based on the protein concentration of each well.

### 2.4 Lipid metabolism

#### 2.4.1 Chemicals and reagent

The mass spectrometry-pure acetonitrile, isopropanol, and chromatography-pure ammonium acetate used in this experiment were purchased from Thermo-Fisher Scientific (FairLawn, NJ, USA). Ultrapure water for the experiments was obtained from Millipore Reference Ultrapure Water System 92 (Billerica, MA, USA) which equipped with a 0.22 μm filter head for liquid-quantity coupling.

#### 2.4.2 Sample preparation

To minimize degradation, samples were thawed under an ice bath. Ten grinding beads were added to each tube of cell samples with 10 µL of deionized water and homogenize for 3 min (BB24, Next Advance, Inc, NY, USA). 300 μL of lipid extraction solvent was added and homogenized again for 3 min. The samples were vortexed and mixed at 1,200 rpm for 20 min at 10°C (MSC-100, Allsheng Instruments, Co. Hangzhou, China), and then centrifuged at 4,000 *g* for 20 min at 4°C (Allegra X-15R, Beckman Coulter, Inc, IN, USA), 20 µL of supernatant was transferred to a 96-well plate and mixed with 80 µL of lipid dilution solvent for LC-MS analysis.

#### 2.4.3 Instrumentation

This project utilized an Ultra High Performance Liquid Chromatography-Triple Quadrupole Mass Spectrometry (UPLC-TQMS) instrument (TACQUITY UPLCXevo TQ- S, Waters Corp. Milford, MA, USA) for targeted lipidomic assays. System optimization and maintenance were performed every 48 h.

#### 2.4.4 Analytical quality control procedures

Endogenous small molecule metabolites are susceptible to changes in ambient temperature and environment, and therefore, sample thawing requires that it be performed slowly on an ice bath slowly, thus avoiding changes in metabolite composition and concentration caused by activation of metabolic enzymes after the sample is sharply returned to room temperature. The reagents used for extraction were pre-frozen and stored in an ice bath. The reagents used for extraction were pre-frozen and stored in a −20°C refrigerator to avoid the exothermic addition of organic solvents to the precipitated proteins, which can lead to the degradation of small molecule metabolites in biological samples. The entire sample preparation process should be completed as quickly as possible. There were reagent blanks and mixed QC samples before and after analyzing each batch of samples. These QCs were also added to monitor the analytical process for possible contamination and data quality.

#### 2.4.5 Sample run order

In order to eliminate errors caused by the order of the analytical process, the samples to be tested were randomized according to the group information, and QC samples, blanks, etc. were interspersed with the overall samples for testing. QC samples, blank samples were interspersed in the overall sample for testing.

#### 2.4.6 Sample control procedure

Samples for each project were entered into Metabo-Profile’s LIMS management system upon receipt. The system assigned a unique identifier, MP ID, that matches the original sample information. This identification was tracked throughout the experiment.

#### 2.4.7 Data analysis

Mass spectrometry-based quantitative metabolomics works by comparing metabolites in a sample of unknown concentration to a set of standard samples of known concentration (quantitative curve) to obtain the actual concentration. A quantitative curve is a curve that analyzes the signal as it varies with the concentration of the measured substance (DUT). For most analyses, a plot of instrument response *versus* concentration will show a linear relationship. This will produce a regression equation of y = aX + *b* where y is the instrument’s corresponding (peak height or peak area), a represents the slope/sensitivity, and b is a constant representing the background signal. The analyte in the unknown sample concentration (X) is calculated from this equation.

### 2.5 Statistical analysis

Data are expressed as mean ± standard error of mean (S.E.M) of each group. Data are expressed as mean ± standard error of mean (S.E.M) for each group. Two-tailed unpaired Student’s t-test was used for comparisons between the two groups, and one-way ANOVA was used to compare the data for CCK8. All statistical analyses were performed using Graphpad Prism 6.0 software (GraphPad software, CA, USA).

## 3 Results

### 3.1 NEFA reduces the viability of hepatocytes in a concentration-dependent manner

**FIGURE 1:**
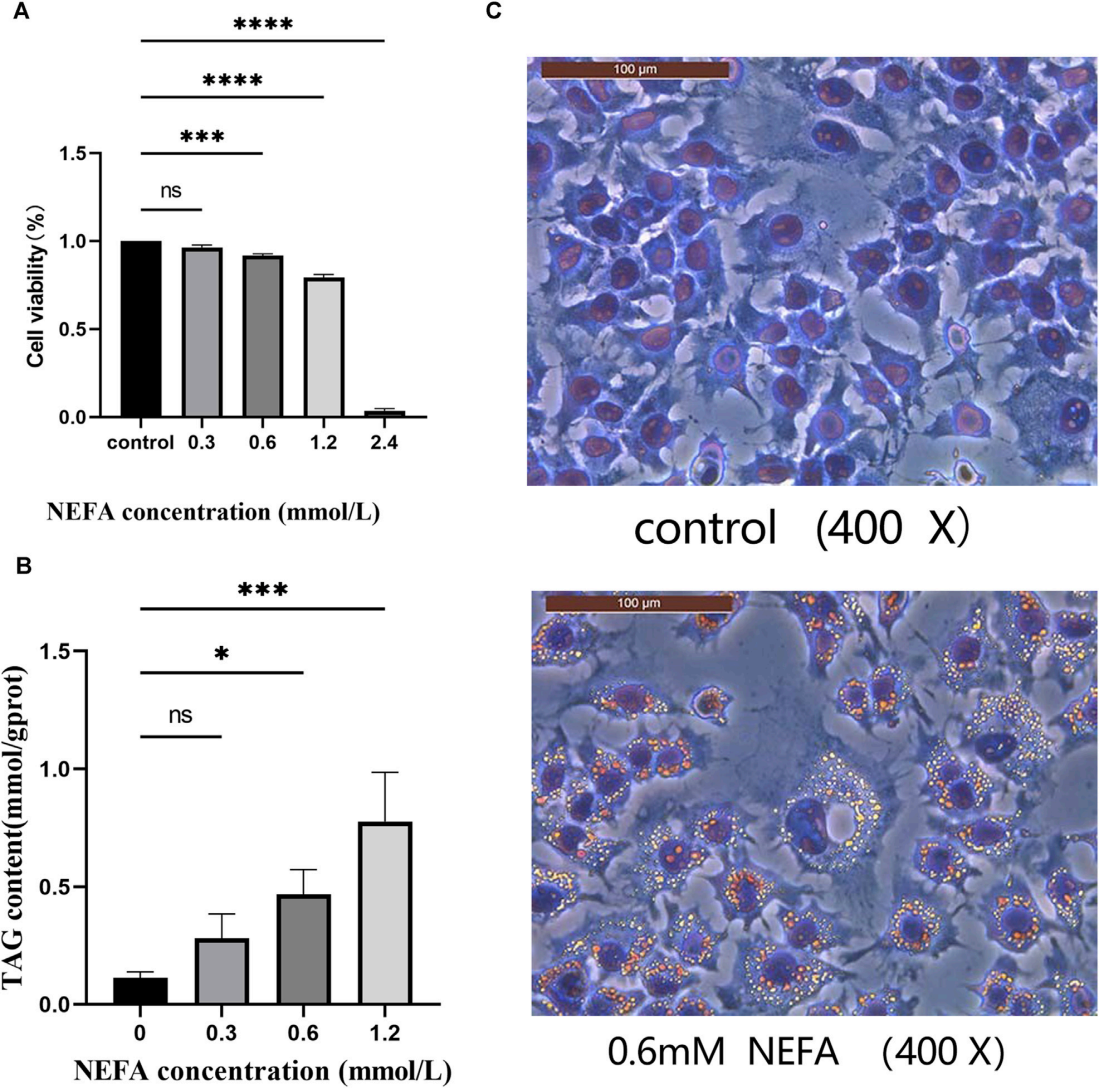
Effect of non-esterified fatty acids (NEFA) on cell viability. **(A)** The cells were treated with different concentrations of NEFA for 12 h, and the cell viability was detected using the CCK8 kit. **(B)** Effects of various concentrations of non-esterified fatty acids (NEFA) on the triglyceride (TAG) content in hepatocytes. **(C)** Oil Red O staining. Data are expressed as the mean ± SEM. *p < 0.05, **p < 0.01, ***p < 0.001, ****p < 0.0001.

In this study, we investigated the toxic effect of NEFA on hepatocytes. The effect of different concentrations (0.3, 0.6, 1.2, 2.4 mM) of NEFA on cell viability was assessed using the CCK-8 assay kit. As shown in Figure 1A, compared with the control group, there was no significant change in cell viability in the 0.3 mM NEFA treatment group. However, the cell viability was significantly decreased after treatment with 0.6 mM NEFA (*p* < *0.001*), 1.2 mM NEFA (*p* < *0.0001*), and 2.4 mM NEFA (*p* < *0.0001*), with 2.4 mM NEFA causing excessive cell damage and reducing cell viability to below 20%. The results revealed that NFEA reduces the cell viability in a concentration-dependent manner.

### 3.2 NEFA promotes lipid accumulation in hepatocytes

The effect of NEFA on lipid accumulation in hepatocytes was analyzed in this study by measuring the TAG contentin cells. As shown in Figure 1B, the TAG content in the 0.3 mM NEFA treatment group did not exhibit a significant change compared to the control group. In contrast, the TAG content was significantly increased in the 0.6 mM (*p* < *0.05*) and 1.2 mM (*p* < *0.001*) NEFA treatment groups compared to the control group. The oil red O staining results also demonstrated that high concentrations of NEFA promote lipid accumulation (Figure 1C).

Consequently, based on the results of CCK-8 and TAG content detection, we chose to employ 0.6 mM NEFA for subsequent experiments.

### 3.3 Effect of NEFA on molecules of lipid metabolism

**FIGURE 2:**
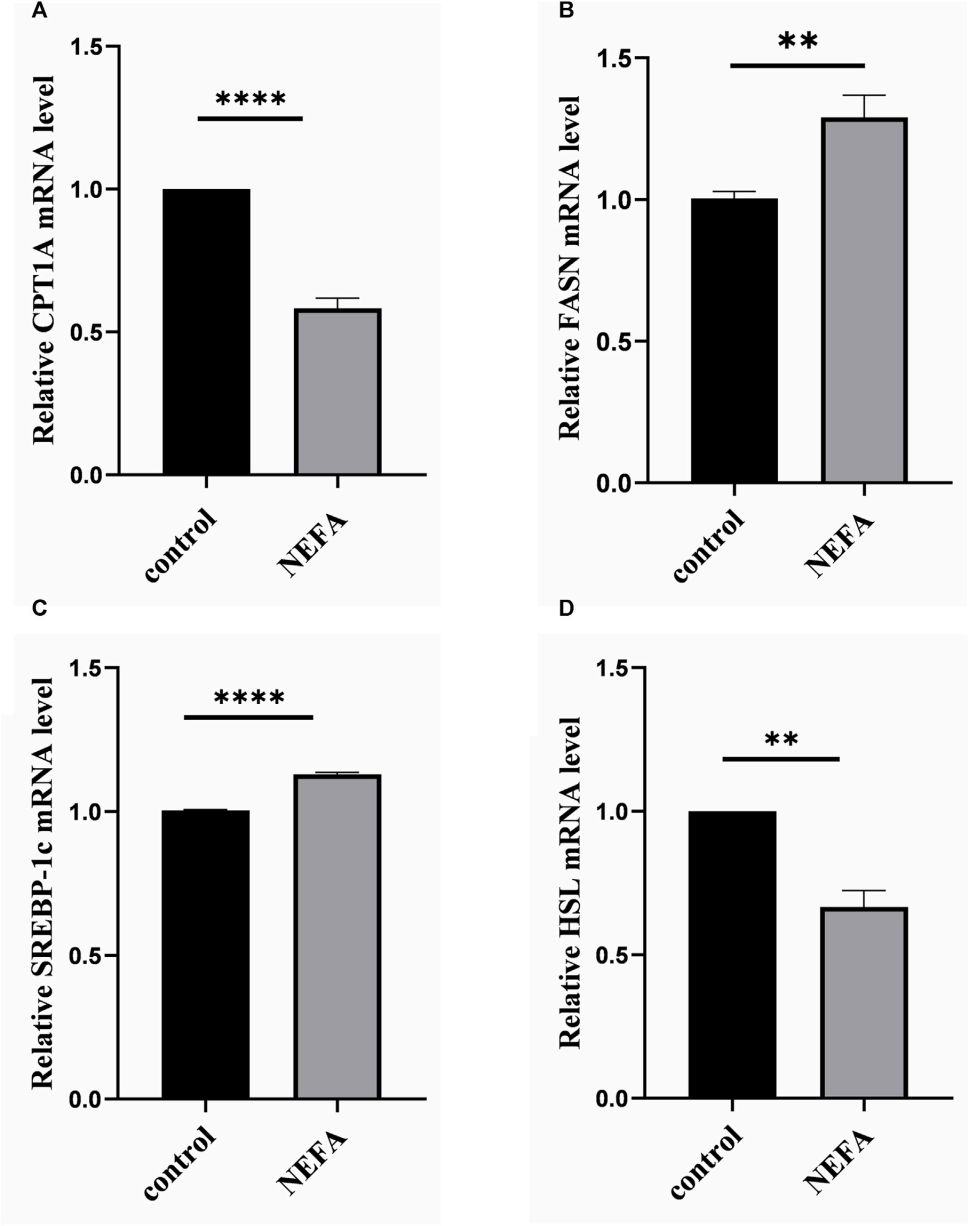
Effects of high NEFA concentration on mRNA expression levels of lipid metabolism-related molecules in hepatocytes. **(A)** CPT1A: carnitine palmitoyltransferase 1 A. **(B)** FASN: Fatty acid synthase. **(C)** SREBP-1c: sterol regulatory element binding protein-1c. **(D)** HSL: hormone sensitive lipase. Data are expressed as the mean ± SEM. *p < 0.05, **p < 0.01, ***p < 0.001.

To further investigate the effect of NEFA on lipid accumulation, this study examined the mRNA expression of key molecules involved in cellular lipid metabolism. Compared with the control group, NEFA treatment significantly increased the expression of lipid synthesis-related molecules FASN and SREBP, and significantly decreased the expression of lipolytic molecules HSL and CPT1A (Figure 2).

In summary, NEFA promotes lipid accumulation in AML-12 cells by regulating lipid metabolism-related molecules.

### 3.4 Exploring the effects of high concentration of NEFA on hepatocytes based on lipidomics

Based on the experimental results presented above, it is evident that high concentration of NEFA can impact the lipid metabolism in hepatocytes, leading to the inhibition of lipolysis and the promotion of lipid accumulation. However, the specific lipid metabolites and metabolic pathways responsible for the disruption of liver cell lipid metabolism induced by high concentration of NEFA remain unclear. Hence, this study employed lipidomics to further elucidate the mechanism underlying the effect of NEFA on the lipid metabolism disorder in hepatocytes.

#### 3.4.1 QC and total sample principal component analysis

**FIGURE 3:**
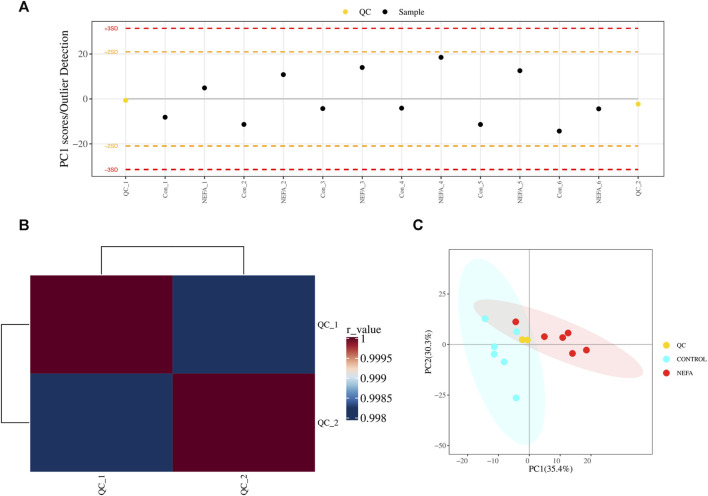
QC and total sample principal component analysis. **(A)** Multivariate control chart: Each point in the chart represents a sample, and any points that exceed three times the standard deviation are considered outliers. **(B)** Pearson correlation analysis: The closer the r-value is to 1, the closer the similarity between the QC samples. **(C)** PCA analysis with QC samples.

Before analyzing the sample data, a portion of the extracted material from 12 test samples was taken to prepare quality control (QC) samples for the subsequent calibration analysis. Multivariate quality control charts were utilized to supervise and judge whether the instrument’s fluctuations are within the normal range. Figure 3A illustrates that the fluctuation of the QC sample was within the range of plus or minus 3 standard deviations, indicating that the instrument’s fluctuations were within the normal range and the data can be used for subsequent analysis. Additionally, as depicted in Figure 3B, Pearson correlation analysis of the QC samples was conducted. A correlation coefficient greater than 0.9 generally indicated good correlation. The experimental results demonstrate that the correlation coefficients between the QC samples are all above 0.9, indicating good experimental repeatability. Furthermore, Figure 3C showed the scores of principal component analysis with QC. The QC samples were closely clustered in the graph, signifying a high degree of clustering and suggesting good stability in instrument detection. Therefore, the data of this model was stable and reliable, and can proceed to the next step of statistical analysis.

#### 3.4.2 Lipid composition analysis

**FIGURE 4:**
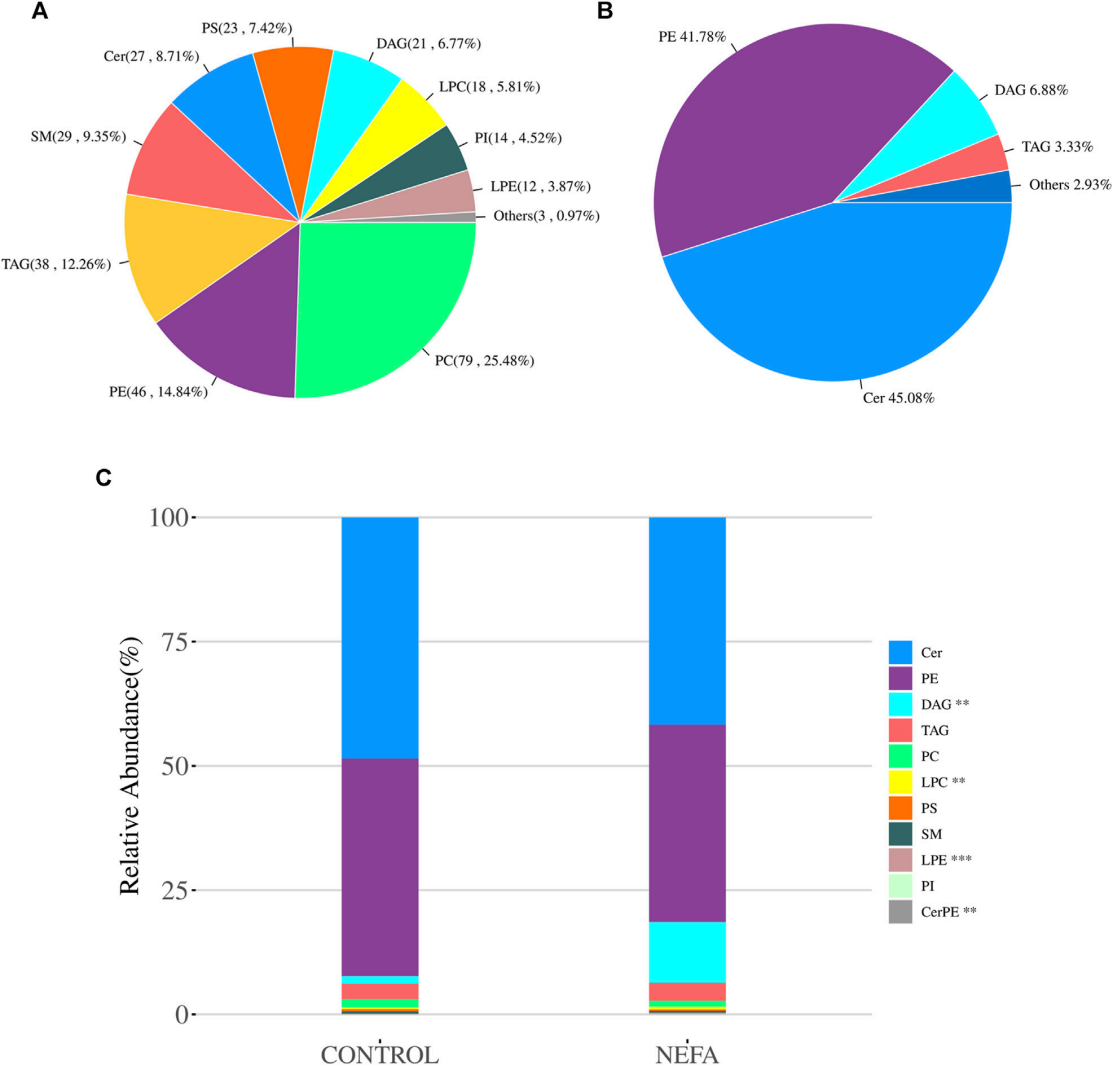
Quality control and principal component analysis of total samples and quantitative analysis of lipidomics. **(A)** Pie chart of average abundency composition of various metabolites in all samples. **(B)** Pie chart of the average abundance composition ratio of each metabolite class in all samples in the NEFA group. **(C)** Stacked bar chart of relative abundance of various metabolites in each group of samples.

After using mass spectrometry to detect the lipids in the samples, a total of 310 metabolites were obtained. These lipid molecules were further analyzed and categorized into 11 subclasses. As shown in Figure 4A, there are 79 types of Phosphatidylcholine (PC), accounting for 25.48% of the total amount of lipid molecules, 46 types of Phosphatidylethanolamine (PE) accounting for 14.84%, 38 types of Triacylglycerol (TAG) accounting for 12.26%, 29 types of Sphingomyelin (SM) accounting for 9.35%, 27 types of Ceramide (Cer) accounting for 8.71%, 23 types of Phosphatidylserine (PS) accounting for 7.42%, 21 types of Diacylglycerol (DAG) accounting for 6.77%, 18 types of lysoPhosphatidylcholine (LPC) accounting for 5.81%, 14 types of Phosphatidylinositol (PI) accounting for 4.52%, 12 types of lysoPhosphatidylethanolamine (LPE) accounting for 3.87%, and the remaining three types of other lipids accounting for 0.97%.

The relative abundance of various metabolites in each sample was shown in Figure 4B. The average abundance of various metabolites in the NEFA group was shown in Figure 4B, with Cer accounting for 45.08%, PE accounting for 41.78%, and DAG and TAG accounting for 6.88% and 3.33% respectively. All quantified lipids in the same group were summed to observe the differences in lipid molecular content among different groups of samples. As shown in Figure 4C, DAG and LPC were significant increase in relative abundance (*p* < 0.01). LPE and CerPE were significant decrease in relative abundance (*p* < 0.001).

#### 3.4.3 Orthogonal partial least squares discriminant analysis

**FIGURE 5:**
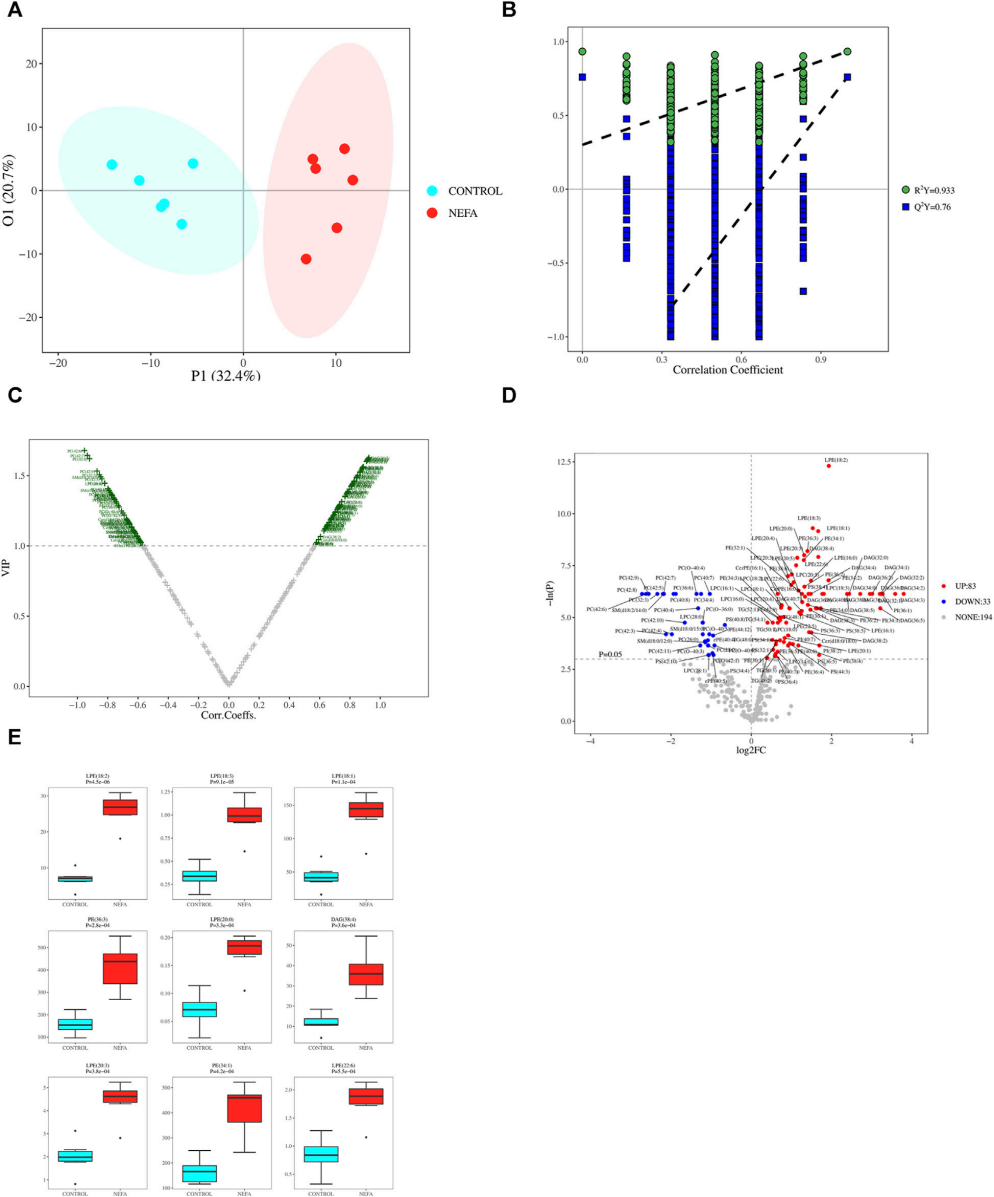
**(A)** OPLS-DA 2D Score Chart. **(B)** OPLS-DA 2D Permutation test Result. **(C)** Metabolite VIP volcano map of OPLS-DA. **(D)** One-dimensional road map of a volcano: Compared to the CONTROL group, the highlighted metabolites in the top right increased in the NEFA group, while the highlighted metabolites in the top left decreased in the NEFA group. **(E)** Boxplot of top 9 differential metabolites ordered by *p*-value.

The PCA model may not be sensitive to variables with low correlations, rendering it unsuitable for identifying differential metabolites. However, this limitation can be addressed by employing OPLS-DA analysis. As illustrated in Figure 5A, the data from the two groups exhibit a clear separation trend, indicating substantial differences between the control and NEFA samples. The OPLS-DA model was validated using Permutation testing. A value of R^2^Y closer to 1 indicates higher model performance, while a Q^2^ value greater than 0.5 signifies model effectiveness. In this case, R2Y was determined as 0.9333 and Q^2^ as 0.76, confirming the validity of the model as illustrated in Figure 5B.

#### 3.4.4 Significant difference lipid metabolite screening

Based on the OPLS-DA model results, volcano plot was used to screen reliable metabolite markers. The volcano plot comprehensively investigated the contribution of metabolites to model grouping (Variable importance in projection, VIP) and the reliability of metabolites (Correlation Coefficients) (Figure 5C). The volcano plot effectively illustrates the distribution and content disparities of metabolites across the samples. Larger absolute values on the horizontal axis indicate greater differences in expression levels between the two sample groups, while larger values on the vertical axis signify more pronounced differences, thus affirming the stability and reliability of the obtained differential lipid metabolites. Using a significance level of *p < 0.05* and an absolute value of log2FC ≥ 0 (FC, Fold Change) as the threshold for screening differential metabolites, 116 metabolites were identified in the comparison between control and NEFA (Figure 5D). Figure 5D reveals that out of the 116 identified metabolites, 83 are upregulated and 33 are downregulated. Boxplot of top 9 differential metabolites ordered by *p*-value were shown in Figure 5E. These metabolites encompass 21 types of DAG, 27 types of Cer, 17 types of PE, 4 types of PS, 12 types of LPE, 18 types of LPC, 79 types of PC, 29 types of PE, 3 types of CerPE, 14 types of PI, 19 types of PS, 29 types of SM, and 38 types of TAG.

**FIGURE 6:**
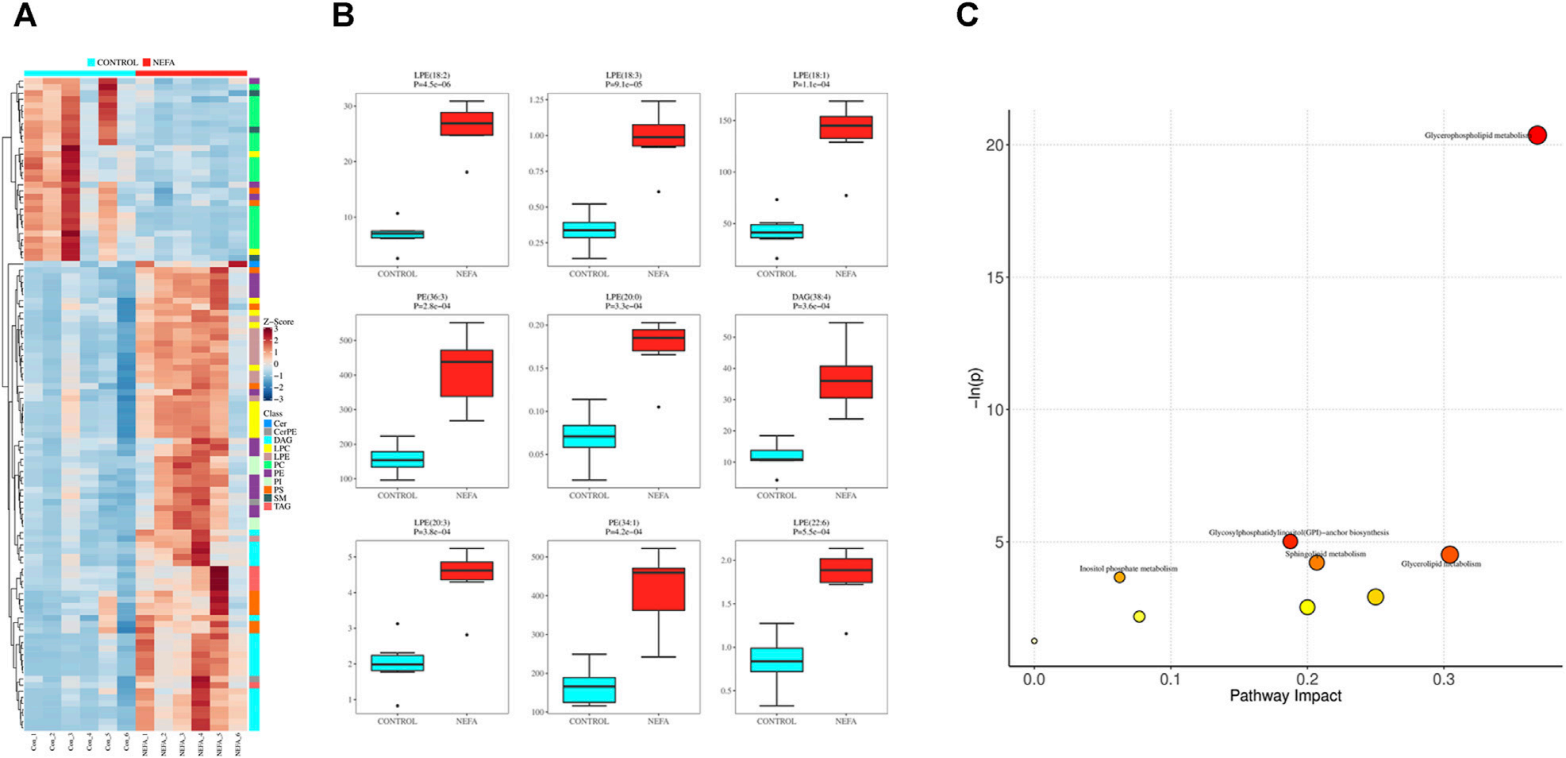
Potential biomarkers and their enrichment pathways. **(A)** Heat map of potential biomarkers. **(B)** Box diagram of potential biomarkers. **(C)** mmu library pathway analysis bubble diagram.

Applying dual criteria (*t*-test *p < 0.05*, |Log2FC|≥0, and OPLS-DA analysis VIP≥1), the selection of potential biomarkers with potential biological significance by combining the different metabolites obtained above is the most reliable approach. This diagram reveals 107 common different metabolites between the two groups, and their distribution in different samples is illustrated in the Figure 6A. Among the 107 potential biomarkers, 77 are upregulated and 30 are downregulated. The boxplot in Figure 6B highlights the 9 most significantly different metabolites: LPE (18:2), LPE (18:3), LPE (18:1), PE (36:3), LPE (20:0), DAG (38:4), LPE (20:3), PE (34:1), LPE (22:6).

Using the selected mmu library for pathway enrichment analysis of differential metabolites, the results were shown in Figure 6C. The differential metabolites enriched in these metabolic pathways are shown in Supplementary Table S1. Each bubble in Figure 6C represented a pathway. The redder the bubble, the closer the *p*-value is to 0, indicating more significant enrichment. The larger the bubble, the more differential lipid metabolites are enriched in that pathway. There were 5 differential metabolic pathways related to lipid metabolism involved in the disordered lipid metabolism process induced by high concentration of NEFA in liver cells, including glycerophospholipid metabolism, glycosylphosphatidylinositol (GPI) -anchor biosynthesis, triglyceride metabolism, sphingolipid metabolism, and inositol phosphate metabolism.

## 4 Discussion

The liver serves as the central organ for lipid metabolism, responsible for regulating the equilibrium of lipids, proteins, and carbohydrates (Mun et al., 2019). In the pathogenesis of fatty liver, the excessive mobilization of adipose tissue due to negative energy balance results in a substantial influx of NEFA into the liver. However, the liver is unable to metabolize excessive NEFA, resulting in its esterification into triglycerides, which then accumulate in the liver (Grummer, 1993; González et al., 2011). This highlights that the disorder in lipid metabolism is the core of fatty liver disease in dairy cows, and numerous studies have been carried out to investigate this phenomenon (Grummer, 1993; Wang et al., 2018; Zhao et al., 2023). Furthermore, the high concentration of NEFA is closely associated with the disorder in lipid metabolism. In healthy cows, the serum NEFA content is lower than 0.4 mM, but the serum NEFA concentration gradually increases to more than 0.5 mM in parturient cows due to metabolic disorders and negative energy balance leading to extensive catabolism of body fat. In cases of particularly severe negative energy balance, the serum NEFA concentration can exceed 1.5 mM (Samiei et al., 2010). Previous research by Dong et al. has revealed that elevated levels of NEFA can stimulate lipid synthesis in the liver, suppress lipolysis, activate inflammatory signaling pathways, and consequently contribute to hepatic lipid accumulation and the initiation of an inflammatory response in cows with fatty liver (Dong et al., 2019). Huang and others discovered that NEFA play a role in mediating the PERK-eIF2α signaling pathway, leading to the upregulation of the key transcription factor SREBP-1c involved in lipid synthesis. This process ultimately promotes lipid synthesis in bovine liver cells. Consequently, their findings indicate that elevated levels of NEFA can impact lipid metabolism in liver cells, contributing to the accumulation of lipids (Huang et al., 2021). The findings of this study suggest that high concentrations of NEFA have the potential to affect the lipid metabolism in liver cells, resulting in lipid accumulation in the cells. Thus, the study seeks to investigate the influence of NEFA on lipid accumulation in liver cells. The results of the cell viability experiment demonstrated that NEFA can significantly decrease cell viability in a concentration-dependent manner. While the cell viability in the 0.3 mM NEFA treatment group did not exhibit significant changes compared to the control group, the cell viability significantly decreased after treatment with 0.6, 1.2, and 2.4 mM NEFA. Notably, treatment with 2.4 mM NEFA resulted in excessive cell damage and significantly reduced cell viability. This suggests that high concentrations of NEFA have the potential to disrupt normal cell metabolism.

Fatty liver in cows is characterized by the significant accumulation of TAG in the liver (Pralle et al., 2021), leading to a substantial impact on its metabolic function (Grummer, 1993). In this study, the TAG content in liver cells from the experimental group (0.6 mM NEFA) showed a significant increase compared to the control group. Zhao (Yang et al., 2023) stimulated primary calf liver cells with 1.2 mM NEFA for 12 h and observed that there was a significant increase in the mRNA expression levels of SREBP-1c, FAS, and ACC1, along with a notable increase in TAG content and lipid droplet content. These results are consistent with our present study, confirming that a high concentration of NEFA can stimulate lipid accumulation in liver cells. However, it is important to note that the aforementioned study employed 1.2 mM NEFA to stimulate primary calf liver cells, whereas our study utilized 0.6 mM NEFA to stimulate AML-12 cells. As different cell types exhibit different tolerances to NEFA stimulation, the final concentration used for stimulation in our study was different from that used in other studies. Furthermore, existing literature (Simino et al., 2021) discusses the *in vitro* model of fatty liver induced by NEFA in AML-12 cells, which supports the credibility of our study.

From the perspective of lipidomics, it is evident that high levels of NEFA are involved in the differential metabolism of lipids in hepatic cells. Moreover, pathway enrichment analysis can help identify differential metabolic pathways, thus elucidating the mechanism by which high levels of NEFA affect lipid metabolism in hepatic cells. This study employed multidimensional and univariate statistical analyses to identify 107 potential biomarkers, a large proportion of which were upregulated lipid metabolites. This suggests that an increase in NEFA level can disrupt lipid metabolism, leading to lipid deposition and lipid metabolism disorders in fatty liver in dairy cows. The most significant differential metabolites encompassed 9 types, primarily belonging to the lipid subclasses of lysophosphatidylethanolamine (LPE), phosphatidylethanolamine (PE), and diacylglycerol (DAG).

By inputting the information of 107 potential biomarkers into the database for further analysis, we obtained 5 different metabolic pathways related to lipid metabolism, including glycerophospholipid metabolism, glycosylphosphatidylinositol (GPI)-anchor biosynthesis, glycerolipid metabolism, sphingolipid metabolism, and inositol phosphate metabolism. The analysis of these metabolic pathways indicates that high level of NEFA may lead to lipid metabolism disorders in liver cells through five differential metabolic pathways. However, the detailed process by which high level of NEFA affect lipid metabolism through these metabolic pathways requires further research. The glycerophospholipid metabolism pathway is significantly involved in the pathogenesis of fatty liver disease. Phosphatidylcholine is the most abundant phospholipid in the body and is an important component of biological membranes, participating in the recognition and signal transduction of membrane proteins. Phosphatidylcholine metabolism is one of the most important components in maintaining the homeostasis of the body (Zhao et al., 2021). The crucial role of phospholipid metabolism in regulating lipid, lipoprotein, and systemic energy metabolism has been extensively proven (Li et al., 2006; Jacobs et al., 2008; Niebergall et al., 2011). The findings of the experiment further confirm the pivotal role of the glycerophospholipid metabolic pathway in the development of fatty liver. Therefore, the targeting of glycerophospholipid metabolism pathway can serve as a potential strategy for managing fatty liver.

In addition, the differences in the metabolism of sphingolipids, glycerides, inositol phosphate, and glycosylphosphatidylinositol anchoring biosynthesis between the 2 cell groups are also very significant. Sphingolipids are a type of lipid that was initially thought to be essential component of organelles and cell membranes. Research has shown that sphingolipid molecules also have biological activities and can participate in the signal transduction of key physiological processes such as cell growth, differentiation, proliferation, migration, and apoptosis (Maceyka and Spiegel, 2014). Therefore, NEFA may affect lipid metabolism disorder through the glycerophospholipid metabolism pathway.

## 5 Conclusion

Therefore, this study aimed to establish an *in vitro* model of fatty liver using NEFA and focus on lipidomics research to identify differential lipid metabolites involved in the pathogenesis of fatty liver.

The results showed that high concentration of NEFA is lipotoxic to cells, promoting lipid accumulation. Further, lipidomics reveals potential metabolites regulated by NEFA LPE (18:2), LPE (18:3), LPE (18:1) via glycerophospholipid metabolism, glycosylphosphatidylinositol (GPI)-anchor biosynthesis, glycerolipid metabolism, sphingolipid metabolism, and inositol phosphate metabolism, indicating their potential regulation role in the pathogenesis of fatty liver.

In summary, this study has provided new insights into the pathogenesis of fatty liver from the perspective of lipid metabolism.

## Data Availability

The datasets presented in this study can be found in online repositories. The names of the repository/repositories and accession number(s) can be found below: https://data.mendeley.com/datasets/h6snst5hm8/1, DOI: 10.17632/h6snst5hm8.1.
