# Supplementary material for: Effect of high NEFA concentration on lipid metabolism disorders in hepatocytes based on lipidomics
Source: Front Pharmacol. 2024 Feb 28;15:1372296. doi: 10.3389/fphar.2024.1372296 (PMC10933074; doi:10.3389/fphar.2024.1372296)
Supplement: Supplementary file 1 [file Table1.docx]

# Supplementary Table

Table 1 Differential metabolites enriched in each differential metabolic pathway

| Metabolic pathways | Differential metabolites |
| --- | --- |
| Glycerophospholipid metabolism | PE(32:1)，PC(26:0)，LPC(16:0)，DAG(32:0)，LPE(16:0)，PS(32:1)，PI(34:1)，PE(32:1)，PC(26:0)，LPC(16:0)，DAG(32:0)，LPE(16:0)，PS(32:1)，PI(34:1)，PE(32:1)，PC(26:0)，LPC(16:0)，DAG(32:0)，LPE(16:0)，PS(32:1)，PI(34:1)，PE(32:1)，PC(26:0)，LPC(16:0)，DAG(32:0)，LPE(16:0)，PS(32:1)，PI(34:1)，PE(32:1)，PC(26:0)，LPC(16:0)，DAG(32:0)，LPE(16:0)，PS(32:1)，PI(34:1)，PE(32:1)，PC(26:0)，LPC(16:0)，DAG(32:0)，LPE(16:0)，PS(32:1)，PI(34:1)，PE(32:1)，PC(26:0)，LPC(16:0)，DAG(32:0)，LPE(16:0)，PS(32:1)，PI(34:1)，PE(32:1)，PC(26:0)，LPC(16:0)，DAG(32:0)，LPE(16:0)，PS(32:1)，PI(34:1)，PE(32:1)，PC(26:0)，LPC(16:0)，DAG(32:0)，LPE(16:0)，PS(32:1)，PI(34:1)，PE(32:1)，PC(26:0)，LPC(16:0)，DAG(32:0)，LPE(16:0)，PS(32:1)，PI(34:1)，PE(32:1)，PC(26:0)，LPC(16:0)，DAG(32:0)，LPE(16:0)，PS(32:1)，PI(34:1)，PE(32:1)，PC(26:0)，LPC(16:0)，DAG(32:0)，LPE(16:0)，PS(32:1)，PI(34:1)，PE(32:1)，PC(26:0)，LPC(16:0)，DAG(32:0)，LPE(16:0)，PS(32:1) |
| Gycosylphosphatidylinositol (GPI) -anchor biosynthesis | PI(34:1)，PE(32:1)，PI(34:1)，PE(32:1)，PI(34:1)，PE(32:1)，PI(34:1)，PE(32:1)，PI(34:1)，PE(32:1)，PI(34:1)，PE(32:1)，PI(34:1)，PE(32:1)，PI(34:1)，PE(32:1)，PI(34:1)，PE(32:1)，PI(34:1)，PE(32:1) |

Table 1 Differential metabolites enriched in each differential metabolic pathway

| Metabolic pathways | Differential metabolites |
| --- | --- |
| Triglyceride metabolism | TG(48:0)，DAG(32:0)，TG(48:0)，DAG(32:0)，TG(48:0)，DAG(32:0)，TG(48:0)，DAG(32:0)，TG(48:0)，DAG(32:0)，TG(48:0)，DAG(32:0)，TG(48:0)，DAG(32:0)，TG(48:0)，DAG(32:0)，TG(48:0)，DAG(32:0)，TG(48:0)，DAG(32:0)，TG(48:0)，DAG(32:0)，TG(48:0)，DAG(32:0)，TG(48:0) |
| Sphingolipid metabolism | SM(d18:0), Cer(d18:0)，SM(d18:0)，Cer(d18:0) |
| Inositol phosphate metabolism | PI(34:1)，DAG(32:0)，PI(34:1)，DAG(32:0)，PI(34:1)，DAG(32:0)，PI(34:1)，DAG(32:0)，PI(34:1)，DAG(32:0)，PI(34:1)，DAG(32:0)，PI(34:1)，DAG(32:0)，PI(34:1)，DAG(32:0)，PI(34:1)，DAG(32:0)，PI(34:1)，DAG(32:0)，PI(34:1)，DAG(32:0)，PI(34:1)，DAG(32:0)，PI(34:1) |
